# Recurrent large earthquakes related with an active fault-volcano system, southwest Japan

**DOI:** 10.1038/s41598-018-32140-8

**Published:** 2018-09-20

**Authors:** Aiming Lin, Peng Chen, Koichiro Sado

**Affiliations:** 1 Department of Geophysics, Graduate School of Science, Kyoto University, Kyoto, 606-8502 Japan; 2 Chiken Sogo Consultants Co. Ltd., Tokyo, 116-0013 Japan

## Abstract

Based on fieldworks, trench excavation, archaeological evidence, and radiocarbon dating ages, we have identified at least three large normal faulting events within Aso caldera in the past ~3000 years, excluding the 2016 M_w_ 7.1 Kumamoto earthquake. These events took place in AD ~1000, BC ~100, and BC ~1100, respectively, suggesting an average recurrence interval of ~1000 years. These events coincide with the timings of three large inferred paleoearthquakes within the Hinagu–Futagawa Fault Zone (HFFZ), where the 2016 Kumamoto earthquake rupture began. On the basis of geological, geophysical, and seismic data, we conclude that the recurrent normal faulting events within Aso caldera were triggered by the active faults of the HFFZ. As for the 2016 Kumamoto earthquake, seismic rupture initiated on the southwest side of the caldera, propagated northeastward, and terminated inside it. These findings demonstrate that large recurring earthquakes within an active fault-volcano system can be studied to improve our understanding of the termination of coseismic rupture propagation, and that the magma chamber beneath Mt. Aso probably hinders the propagation of coseismic rupture during large earthquakes.

## Introduction

**Figure 1:**
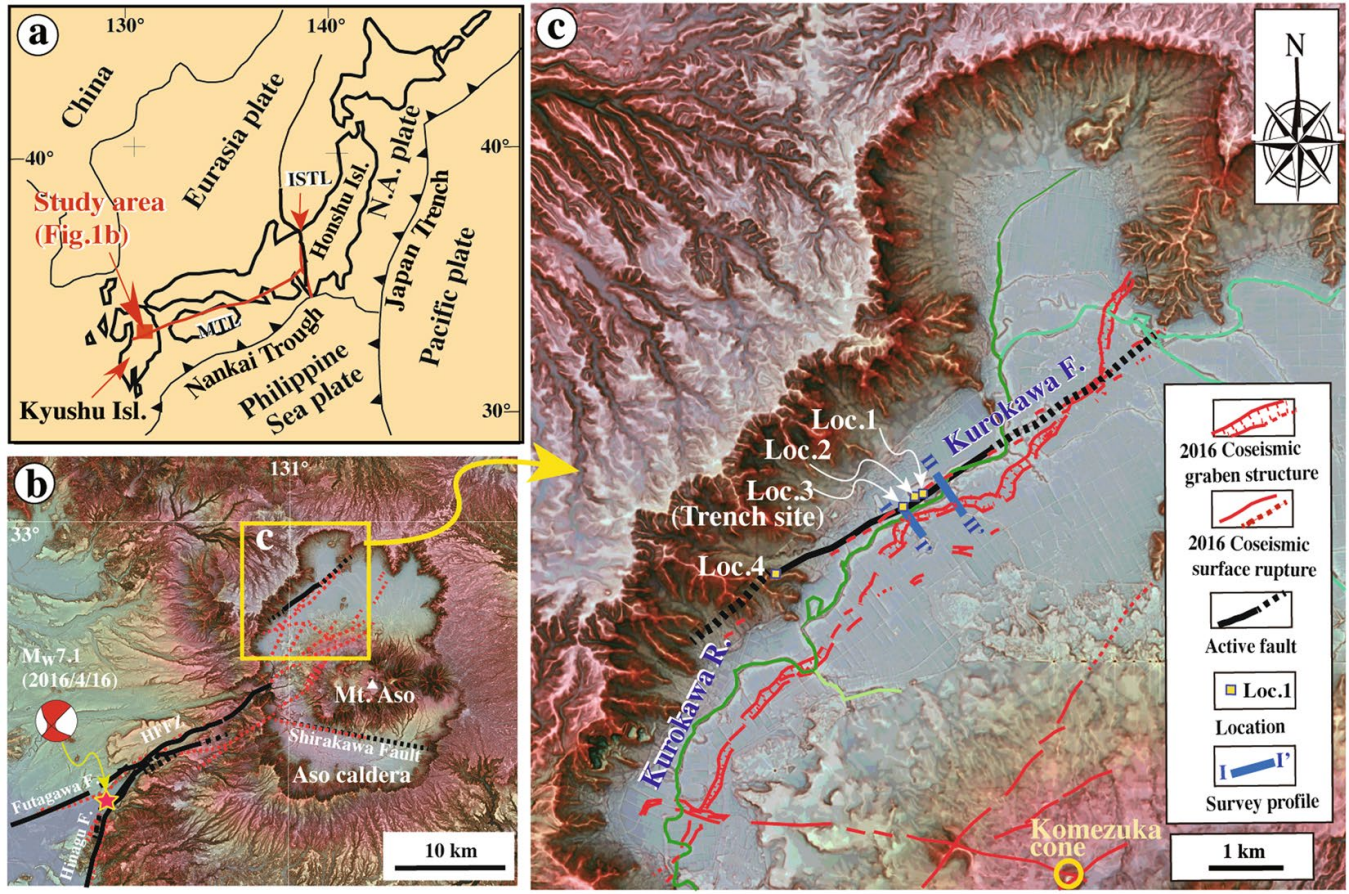
Maps of the study area. (**a**) Index map of the study area showing the tectonic setting. (**b**) Red-shaded relief map showing the distribution of coseismic surface ruptures produced by the 2016 Kumamoto earthquake along the HFFZ. (**c**) as in (**b**), but for the area within Aso caldera (coseismic surface rupture data are from previous studies^3,4^). Active fault data are from RGAFJ^37,38^ and Geographical Survey Institute^39^. Epicenter data and focal mechanisms are from National Research Institute for Earth Science and Disaster Resilience^40^. I-I’ and II-II’: Survey files of electrical resistivity and electromagnetic measurements; MTL: Median Tectonic Line; ISTL: Itoigawa–Shizuoka Tectonic Line; Honshu Isl.: Honshu Island; Kyushu Isl.: Kyushu Island; HFFZ: Hinagu–Futagawa fault zone; Hinagu F: Hinagu Fault; Shirakawa F: Shirakawa Fault; Kurokawa F: Kurokawa Fault. Kurokawa R.: Kurokawa River.

**Figure 2:**
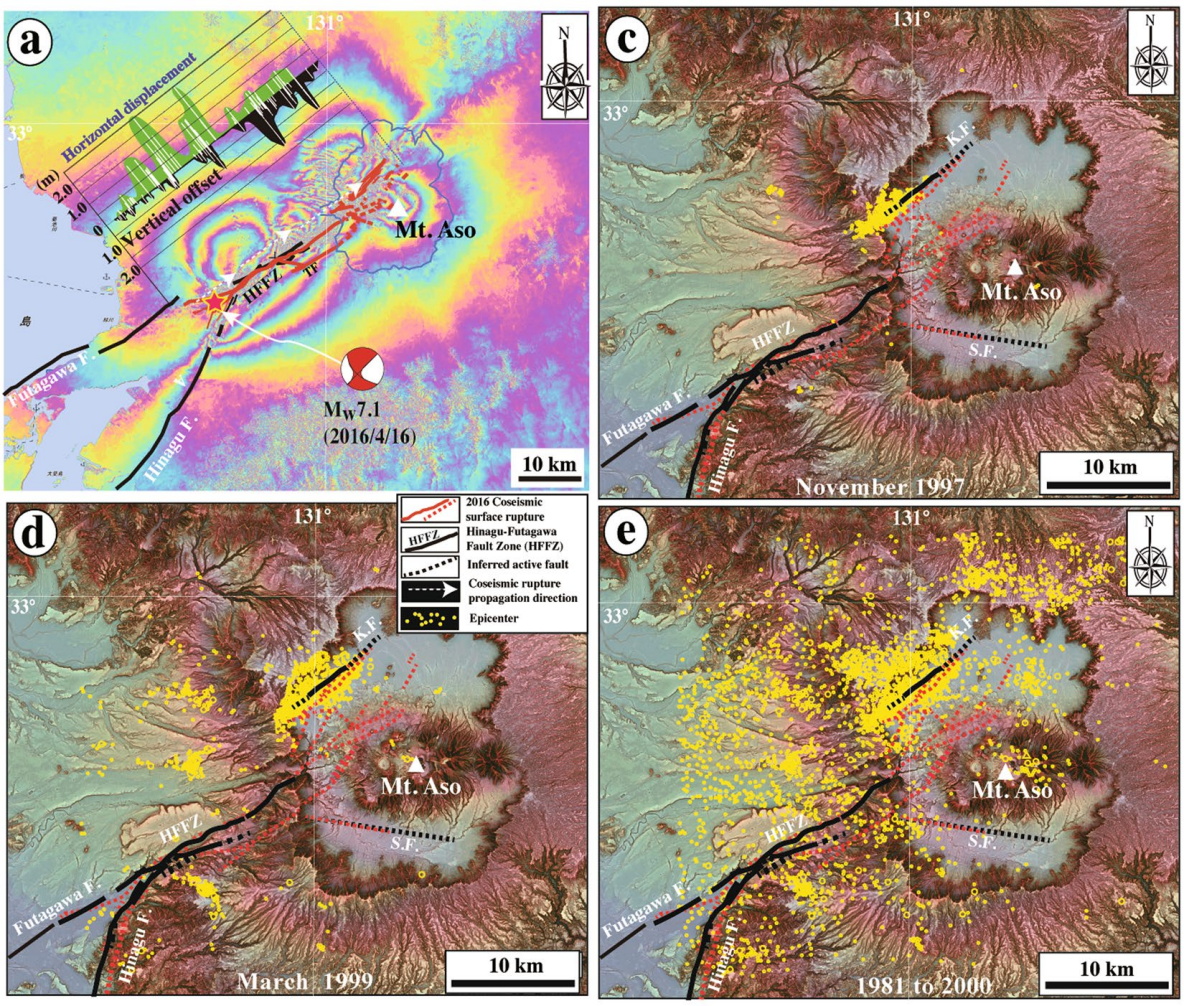
Ground deformation caused by the 2016 Kumamoto earthquake and seismicity in the area around the study region. (**a**) InSAR image showing the ground deformation, generated from PALSAR-2 data acquired on 16 January 2016 and 20 April 2016 [modified from Geospatial Information Authority of Japan (GSI)]^41^. Also shown is the distribution of displacement along the coseismic surface rupture zone^41^. Surface displacement distribution (data are from Lin^4^) and deformation features caused by the 2016 Kumamoto earthquake are indicated (data from Geospatial Information Authority of Japan^41^). Active fault data are from RGAFJ^37,38^ and Geographical Survey Institute^39^. Epicenter data and focal mechanism are from National Research Institute for Earth Science and Disaster Resilience^40^. Hinagu F: Hinagu Fault; Futagawa F: Futagawa Fault. (**b–d**) Red-shaded relief maps showing seismicity in the area around Aso caldera. Seismic data are from Sudo and Ikebe^10^. K.F.: Kurokawa Fault; S.F.: Shirakawa Fault. (**c**,**d**) Earthquake swarms occurred in November 1997 (97/11/1-97/12/1, total number 138) and March 1999 (99/1/8-2000/3/21, total number 767)^10^, respectively. (**e**) All earthquakes (total number 3655) occurred in the period 1981/4/1–2000/3/21^10^.

Large recurring earthquakes generally occur on mature, active faults, and often accompany or precede volcanic eruptions^1–8^. Previous studies reveal that the 2016 M_w_ 7.1 Kumamoto earthquake produced a ~40 km long coseismic surface rupture zone, mostly along the pre-existing active faults of the Hinagu–Futagawa Fault Zone (HFFZ); the rupture zone extends into Aso caldera in central Kyushu Island, southwest Japan^3,4,9^ (Figs 1 and 2a). The newly formed coseismic ruptures under Aso caldera are considered to be potential new channels for magma venting, and these ruptures have changed the spatial heterogeneity and other mechanical properties of Aso volcano^3^. After the 2016 Kumamoto earthquake, Aso began to erupt on 8 October 2016 after 36 years of dormancy, suggesting a close relationship between volcanic eruptions and faulting in this case^4^.

In this study, to better understand the relationship between the paleoseismicity and fault-volcano system structure, we conducted a comprehensive study of the active faults of the HFFZ–Aso volcano system.

## Methods

This study is mainly based on field and trenching investigations, and radiocarbon dating ages for paleoseismic analyses, and geophysical surveys using electrical and electromagnetic methods, and seismic array observations for analyzing fault structures. The trench was excavated across the coseismic surface rupture produced by the 2016 M_w_ 7.1 Kumamoto earthquake. Radiocarbon dating of samples was performed by accelerator mass spectrometry (AMS) by using bulk organic soils which were pretreated for excluding the modern plant carbon based on the Standard Pretreatment Protocols at Beta Analytic, USA. Two electrical and electromagnetic profiles were measured across the 2016 coseismic surface rupture zone (Profiles I-I’ and II-II’ shown in Fig. 1b). The electromagnetic measurements were carried out using an audio-magnetotelluric (AMT) method.

### Seismotectonic setting

Mount Aso, Japan is one of the largest active volcanoes in the world, with a caldera area of 380 km^2^. Volcanic activity in the Aso volcanic cluster started at ~0.3 Ma with a large eruption that produced pyroclastic flows. Subsequently, four large eruptions produced pyroclastic and lava flows across a wide area, including the study area (Fig. 1)^10–13^. The Aso volcanic cluster comprises seven craters including Nakadake cone, which is the largest volcano within the caldera, and Komezuka and Kishima cones, which were ruptured by the 2016 M_w_ 7.1 Kumamoto earthquake^3,4^ (Figs 1 and 2a). The basement rocks are mainly Paleozoic metamorphic rocks, and marine sediments of the Mifune Group formed in 82–93 Ma^13^.

Two active faults have developed within Aso caldera; the NE-striking Kurokawa Fault (part of which was previously called Nijutoge Fault^10^) developed mostly along the Kurokawa River, on the western side of the caldera (Fig. 1b,c), is the target of this study, and the WNW-striking Shirakawa Fault. Within the caldera, coseismic surface ruptures related to the 2016 Kumamoto earthquake, that cut throughout the southwestern ring of the caldera, developed mainly along the Kurokawa Fault and are typically associated with coseismic graben structures^3,4^ (Fig. 1b). The WNW-striking Shirakawa Fault cuts alluvial fans that were formed by southward-flowing tributaries of the Shirakawa River. The 2016 coseismic surface ruptures formed also along the fault scarp of the Shirakawa Fault over a distance of ~7 km^4^ (Figs 1b and 2b–d). Recent seismicity, including the 1997 and 1999 earthquake swarms within Aso caldera, is mainly concentrated along the Kurokawa Fault (Fig. 2b–d)^10^. Seismic data indicate that the Kurokawa Fault is an active seismogenic fault that produces volcanic earthquakes within Aso caldera, which are mainly associated with normal faulting and volcanic activity due to a crustal heat source, i.e., magmatic material^14^.

On the southwest side of Aso caldera, two other main active strike-slip faults have developed along the topographic boundary between the mountain to the southwest side and the Kumamoto Basin to the northeast: the Hinagu Fault strikes NNE–SSW to NE–SW and extends for ~81 km, while the Futagawa Fault strikes NE–SW to ENE–WSW and extends for ~64 km^15^. These faults form a continuous fault zone called the Hinagu-Futagawa Fault Zone (HFFZ; Fig. 1b). The 2016 M_w_ 7.1 Kumamoto earthquake produced a ~40 km long surface rupture zone that initiated at the epicenter, ~30 km southwest of Aso caldera, propagated northeastward along the pre-existing HFFZ into the caldera, and finally terminated inside it^3,4^ (Figs 1b and 2a). Recent study showed that the recurrence interval of large earthquakes on the HFFZ is ~1000 years and the average strike-slip rate is ~1–2 mm/yr^16^.

### Graben structures within Aso caldera

**Figure 3:**
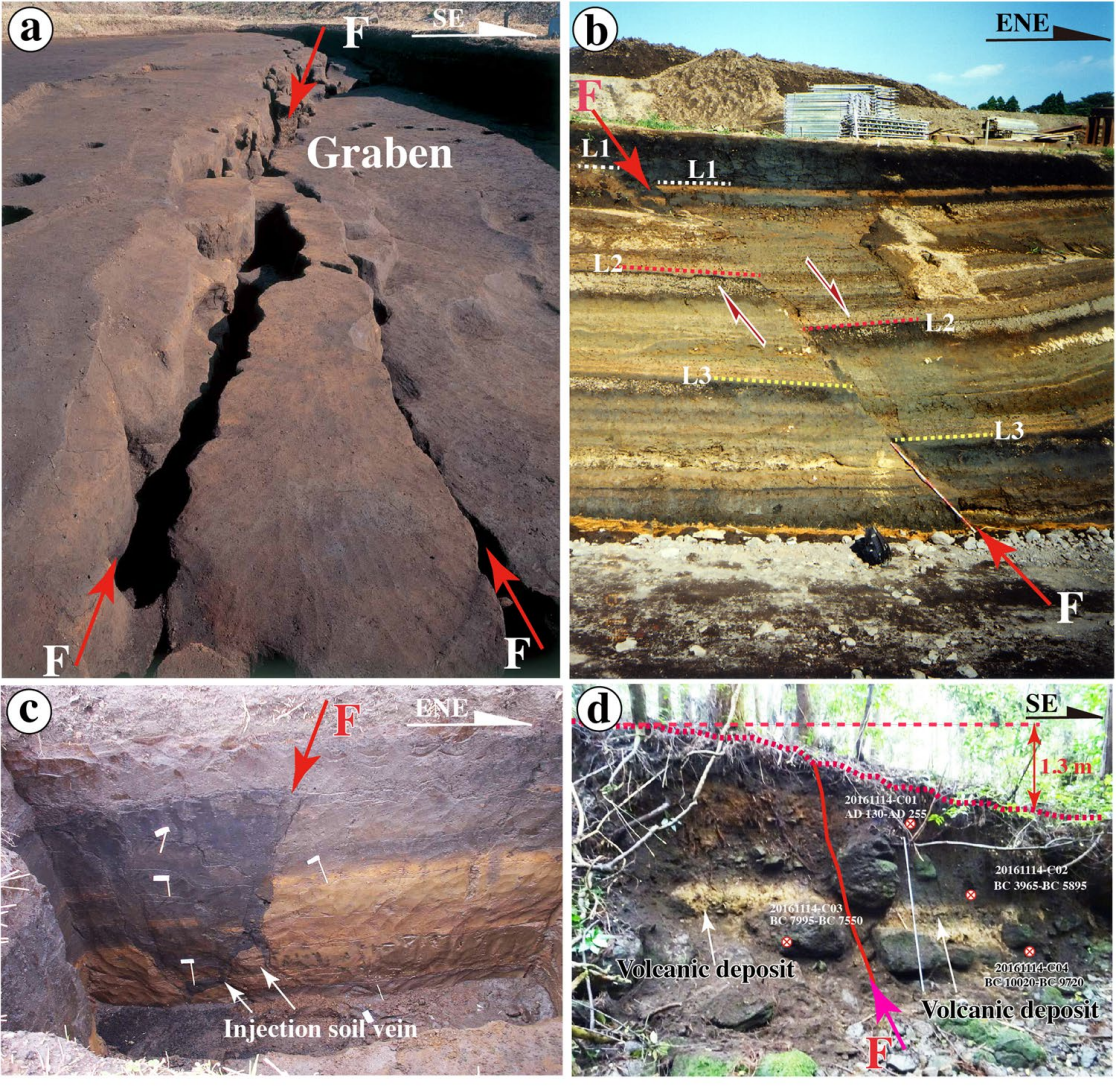
Representative photographs of the Kurokawa Fault, showing normal fault structures. (**a**) Graben structures exposed at an archaeological excavation site (Loc. 1, modified from EDKP^17^). (**b**,**c**) Normal fault exposed at a waterway construction site (Loc. 2). The near-surface soil layer was vertically offset by ~0.7 m in (b); in contrast, layers L2 and L3 are vertically offset by ~2 m, indicating the accumulated offset along the fault (F) (**b,c**) photograph courtesy Y. Sudo; b,c: modified from Sudo and Ikebe^10^). (**c**) Near-surface dark soil material has been injected in the yellowish sandy soil layer along the fault (F). (**d**): Fault outcrop at Loc. 4, where the near-surface soil and alluvial deposits are offset. A 2 m long measuring tape in the center of the photograph is shown for scale. See Table 1 for ^14^C dating results.

**Figure 4:**
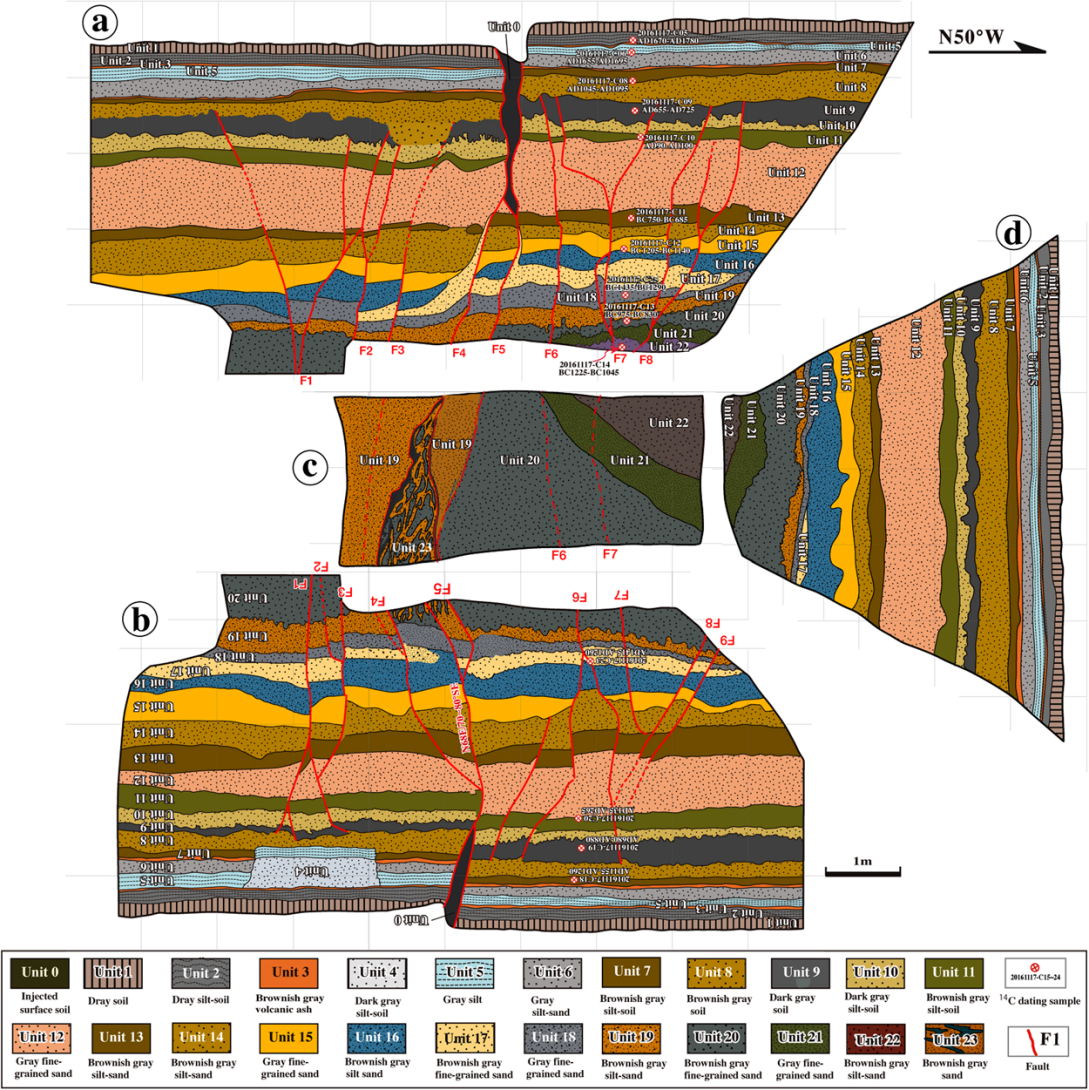
Sketches of the exposed walls in Trench A. (**a**) Southwest wall, (**b**) northeast wall, (**c**) floor of the trench, and (**d**) northwest wall. See Table 1 for ^14^C dating results.

**Figure 5:**
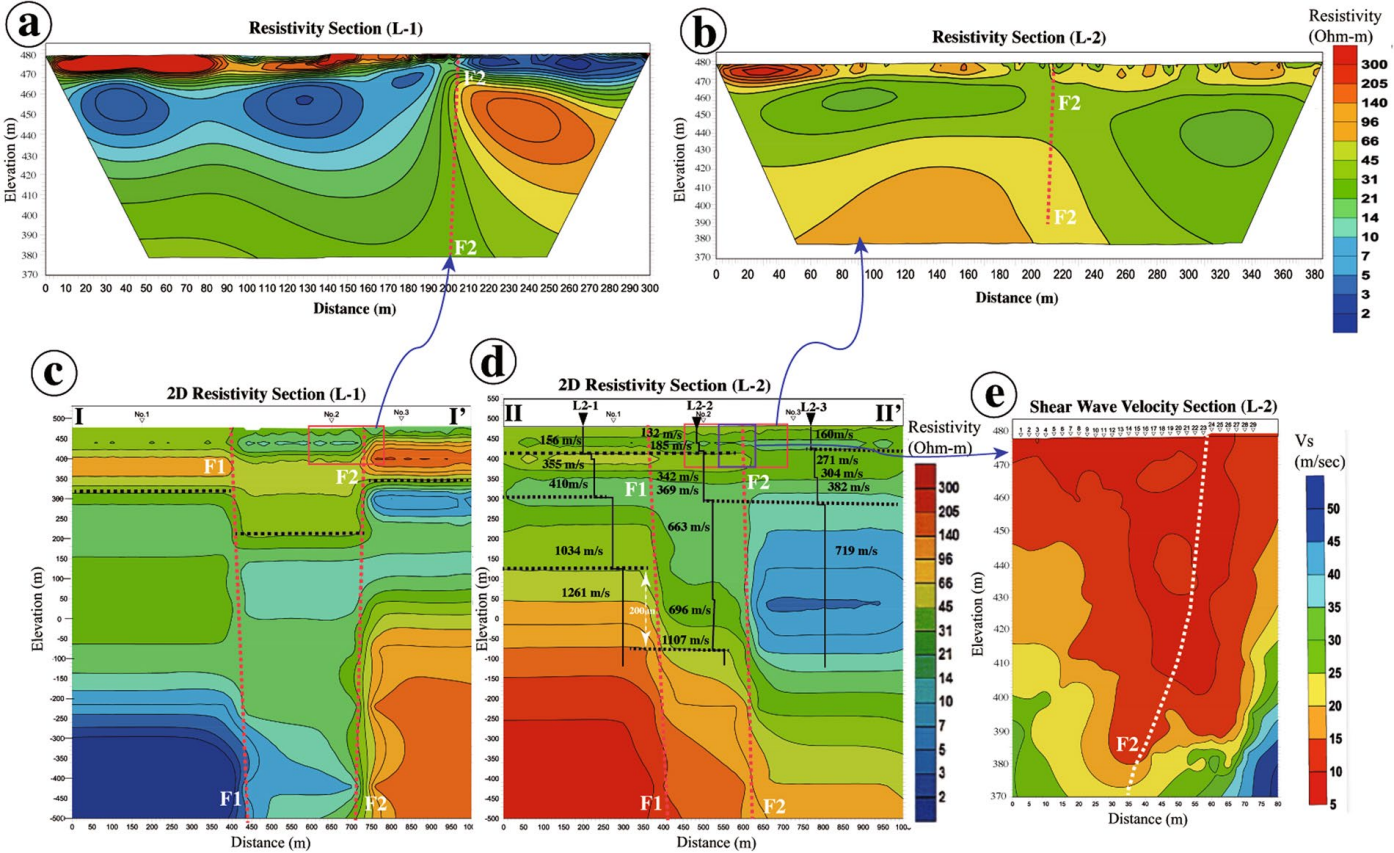
Electrical resistivity (**a**,**b**) and electromagnetic (**c**,**d**) profiles I-I’ and II-II’ across the Kurokawa Fault, and (**e**) array profile showing the S-wave velocity structures of near-surface sedimentary layers. L2-1~L2-3: Microtremor array survey stations. S-wave velocities show that the lower depth of ~1000–1100 m/s stratigraphic layer is offset by ~200 m in vertical (**d**). See Fig. 1 for detail locations of profiles I-I’ and II-II’.

Coseismic graben structures have developed along the coseismic surface rupture zones produced by the 2016 Kumamoto earthquake on the western side of Aso caldera. These grabens extend for ~10 km, mostly along the Kurokawa Fault, with a maximum vertical offset of 1.75 m^3,4^ (Fig. 1b,c and Supplementary Fig. S2). The Kurokawa Fault is characterized by graben structures that were observed in trenches and fault outcrops at Locs. 1–4 (Figs 2–4) and are identified from resistivity and electromagnetic profiles across the fault zone (Fig. 5). Normal faults with vertical offsets of up to ~3 m were observed in a large-area excavation site (>1 × 10^4^ m^2^) around Locs. 1 and 2 that was part of an archaeological study of Yayoi ruins^10^. Many fractures are found at the trench site in both horizontal excavation sections and trench walls, and they cut the near-surface soil layers and form graben structures (Fig. 3a–c and Supplementary S2a,b). Archaeological remains have been unearthed in a graben structure (1–10 m wide) at the site, including pottery, jade, and stone implements from the Yayoi period^17^ (BC ~300 to AD ~300; see Supplementary Fig. S2c,d).

At Loc. 2, a waterway construction site on the Kurokawa River located ~100 m northeast of Loc.1, a fault striking N60°E and dipping ~60° to the southwest was observed at an outcrop with length >20 m and height ~10 m (Fig. 3c)^10^. The fault cuts near-surface sedimentary layers of sandy soil and volcanic deposits, in which dark surface soil materials have been filled in fractures of the fault zone (Fig. 3b,c)^10^. Based on the deformation features of the sedimentary layers and radiocarbon dating ages, the most recent faulting event at this site is estimated to have occurred ~1000 years ago^10^.

At Loc. 4, we found a normal fault at the boundary between the mountain and the lowland area (Figs 1c and 3d). A 1.3 m high fault scarp occurs on an alluvial fan that was produced by a southward-flowing tributary (Fig. 3d). The alluvial deposits comprise a yellowish volcanic ash layer, dark gray near-surface sandy soil materials, and alluvial sand-gravel that contains large gravels (>1 m in size), which are cut by a fault striking N60°E and dipping SE at an angle of ~70° (Fig. 3d). Radiocarbon dating of materials from the near-surface soil and the sandy-soil deposit overlying the volcanic ash layer yields ages of 1810 yr BP and 5100 yr BP, respectively (Fig. 3d, Table 1), indicating that at least one normal faulting event occurred in this area over the past ~1800 yrs.

**Table 1:** Radiocarbon dating ages for samples collected from trench (Loc. 3) and outcrop (Loc.4).

Sample code	Unit no^*^	Laboratory ID^†^		Sample material	Radiocarbon age^‡^ (BP)			Calibrated date^§^ (2σ uncertainties)
20161114-C01	Outcrop	Beta-	450968	organic sediment	1810	±	30	AD 130–255
20161114-C02	Outcrop	Beta-	450969	organic sediment	5100	±	30	BC 3965–3895
20161114-C03	Outcrop	Beta-	450970	organic sediment	8560	±	30	BC 7595–7570
20161114-C04	Outcrop	Beta-	450971	organic sediment	10150	±	30	BC 10020–9760
20161117-C05	2	Beta-	450972	organic sediment	130	±	30	AD 1670–1780
20161117-C06	4	Beta-	450973	organic sediment	180	±	30	AD 1655–1695
20161117-C08	7	Beta-	450975	organic sediment	880	±	30	AD1045–1095
20161117-C09	9	Beta-	450976	organic sediment	1310	±	30	AD 655–725
20161117-C10	11	Beta-	450977	organic sediment	1830	±	30	AD 90–100
20161117-C11	13	Beta-	450978	organic sediment	2430	±	30	BC 750–685
20161117-C12	15	Beta-	450979	organic sediment	2900	±	30	BC 1205–1140
20161117-C13	20	Beta-	450980	organic sediment	2760	±	30	BC 975–830
20161117-C14	23	Beta-	450981	organic sediment	2940	±	30	BC 1225–1045
20161117-C18	7	Beta-	464025	organic sediment	840	±	30	AD 1155–1260
20161117-C19	9	Beta-	464026	organic sediment	1240	±	30	AD 680–880
20161117-C20	11	Beta-	464027	organic sediment	1790	±	30	AD 135–265
20161117-C23	17	Beta-	464028	organic sediment	3070	±	30	BC 1415–1260
20161117-C25	18	Beta-	450982	organic sediment	3110	±	30	BC 1435–1290

^*^Unit.no is corresponding to that shown in Fig. 3 and stated in the text concerned^**†**^ . Samples were analyzed at Beta Analytic Inc., USA (Beta No) via accelerator mass spectrometry (AMS).

^‡^Radiocarbon ages were measured using AMS (Accelerator Mass Spectrometry) and are referenced to the year AD 1950. Analytical uncertainties are reported at 2σ.

^§^Dendrochronologically calibrated calendar age by Method A from CALIB Radiocarbon Calibration Version 6.1 (Stuiver *et al*.^42^.).

Analysis of resistivity and electromagnetic profiles across the coseismic surface rupture zone and the Kurokawa Fault reveals that the graben structure lies within a zone that is 200−300 m wide, with sharp resistivity boundaries along two main steeply-dipping (>80°) normal faults that cut the near-surface sedimentary deposits and extend to depths of >1 km (Fig. 5a–d). S-wave velocities estimated from array microtremor surveys suggest that the lower depth of ~1000–1100 m/s stratigraphic layer is ~120 m above sea level on the NW side of the F1 fault and ~70 m below sea level on the SE side (Fig. 5d). Analysis of resistivity profiles and S-wave velocities measured *in situ* indicate that the vertical offset of layers with the same resistivity and velocity values varies from 30 m in near-surface layers at depths of <100 m, and up to ~200 m in the layers at a depth of >500 m along the F1 fault within the graben structure (Fig. 5c,d). The maximum coseismic vertical offset measured at the trench site along the 2016 surface ruptures is <0.5 m^3,4^ (see Supplementary Fig. S3). The deformation features of the strata show that apparent vertical offsets of up to ~200 m have accumulated in the sedimentary sequence, owing to the many large normal-faulting earthquakes within the caldera similar to those caused by the 2016 M_w_ 7.1 Kumamoto earthquake.

### Trench investigations

A trench was excavated across the coseismic rupture zone at Loc. 3, ~200 m southwest of Loc. 2, along which the topographic surface is vertically offset by 0.3–0.5 m (Fig. 1c and Supplementary Fig. S3). The trench walls were sketched using a 1.0 m grid overlay (Fig. 4 and Supplementary Fig. S4) and are described in detail below. 14 soil samples containing organic materials were taken from this trench for radiocarbon dating. Dendrochronologically-calibrated calendar ages were obtained using the calibration method^16^. The dating results and calibrated ages are listed in Table 1.

The deposits exposed in the trench include sandy soil, volcanic ash deposits, and fine-grained sand-silt that can be divided into 24 sedimentary units (Units 0–23, Fig. 4) based on the properties, color, and layering structures of the sediments. Units 0–8 consist of soil materials and volcanic sediments containing organic soils that are brownish-gray to dark gray in color and yield calibrated ^14^C ages of AD 1045–1670 (Fig. 4, Table 1). Unit 9 is composed of dark-gray soil materials and yields ^14^C ages of AD 655–880. Units 10–22 consist of silt to fine-grained sand sediments with some dark gray soil materials, yielding ^14^C ages of BC 1435 to AD 100 (Fig. 4, Table 1). All of these sedimentary layers are cut by faults F4 and F5. The sedimentary layers of Units 9–23 are cut by faults F1–F3 and F6–F9, and overlain by the sedimentary layers of Units 1−8 (Fig. 4). Liquefaction structures are observed in the sand sediment layers of Units 17–19 and Units 22–23 (Fig. 4, see Supplementary Figs S4 and S5).

### Identification of morphogenic faulting events

Based on the structural features, including fault structures, sedimentary sequences, and deformation features of the sediment layers as well as radiocarbon dating age data, we identified at least three large earthquakes (E2–E4) in the past ~3000 years that occurred prior to the 2016 M_w_ 7.1 Kumamoto earthquake (E1) on the Kurokawa Fault. These are discussed below.

**Figure 6:**
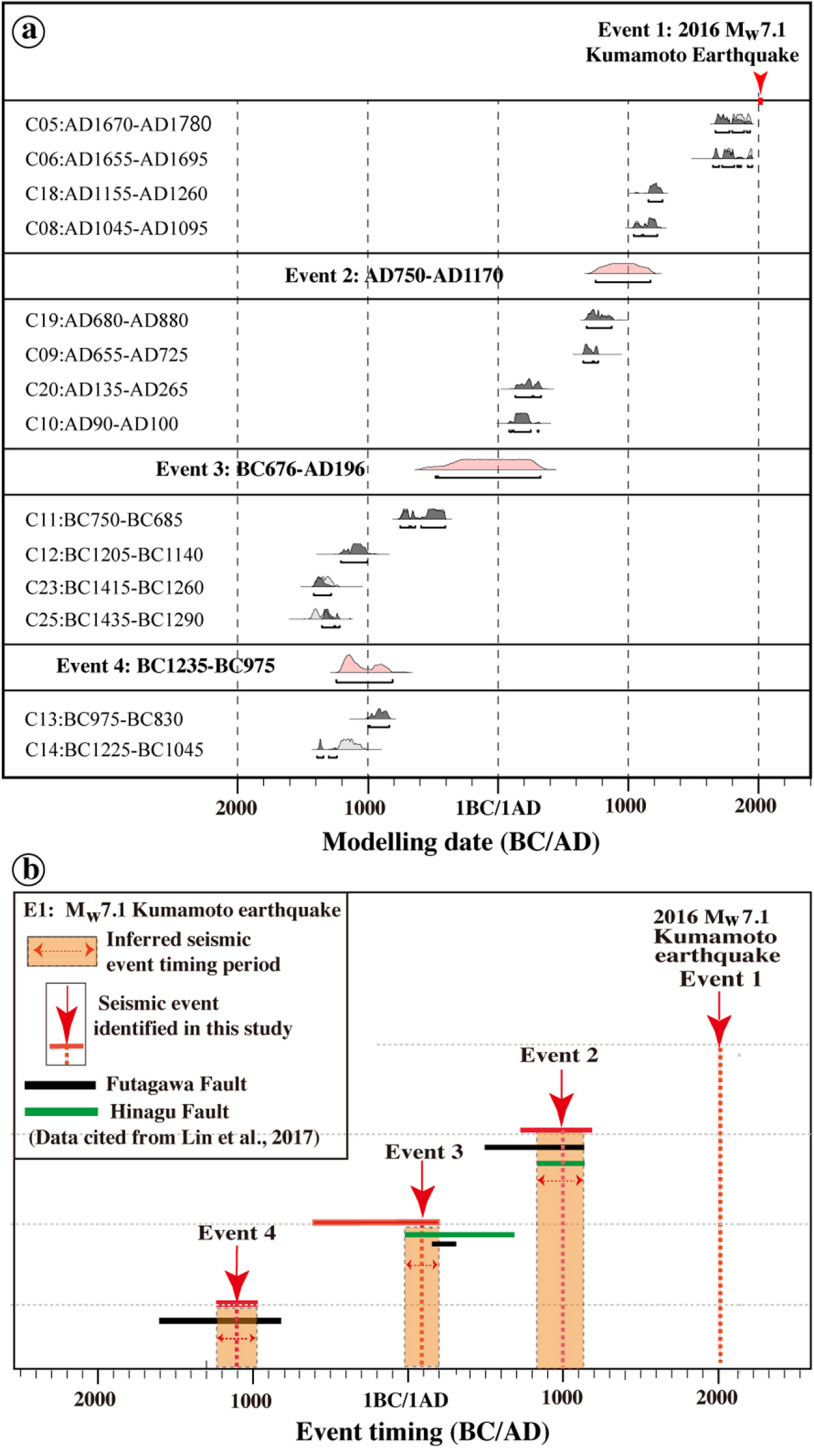
Bayesian model of paleoearthquake dates and inferred timing of faulting events. (**a**) Bayesian model of paleoseismic event timing, made using OxCal version 4.2^43^. Probability distribution functions (PDFs) for radiocarbon dating samples are shown in light gray and posterior PDFs are shown in dark gray. Modeled earthquake ages are shown as PDFs and labeled by event number. Lines below each distribution indicate the 95.4% confidence ranges. (**b**) Event dates including the 2016 M_w_ 7.1 Kumamoto earthquake (Event 1) and three late Holocene historic/paleoearthquakes (Events 2–4) identified in this study from offset features and deformed alluvial sediments in Trench A from the previous study^16^.

All of the sedimentary layers exposed in the trench were offset by faults F4 and F5, and a vertical offset of ~0.3 m, related to the 2016 Kumamoto earthquake (E1), was observed at both the ground surface and in the sedimentary layers (Fig. 4). The sedimentary layers of Units 9–23 are cut by faults F1–F3 and F6–F9, which are overlain by Units 0–8 (Fig. 4). These observations indicate that a faulting event occurred after the deposition of Unit 9 (AD 655–725, AD 680–880) and before Unit 8 (AD 1045–1095, AD 1155–1260), coincident with the normal faulting event inferred at Loc. 2, where soil veins filled in fractures within the near-surface sedimentary layers at ~1000 years ago^10^ (Fig. 3). Fault scarps at Loc. 4 are vertically offset by ~0.5–1.0 m, indicating a normal faulting event in the past 1800 years (Fig. 3d). Based on the timing constraint model, we infer that a faulting event (E2) occurred in the period between AD ~800 and AD ~1200 (Event 2 in Fig. 6a).

The penultimate faulting event (E3) occurred during the deposition of units 11 and 12. Fine-grained sand material was injected upward into the sand layer of Unit 12 along faults F4 and F5, these injection veins can be traced to the sand layers of Unit 17 with irregular boundaries (Fig. 4; see Supplementary Fig. S4d–g). These upward-injected sand veins are in turn cut by the downward injection of veins of dark-gray surface soil formed along faults F4 and F5 by the 2016 Kumamoto earthquake (Fig. 3; see Supplementary Figs S4d and S5). Furthermore, the sedimentary layers of Units 15–17 were offset ~0.3–0.5 m along F4 fault (Fig. 4b and Supplementary Fig. S4f). These structural features show that the sand material of Unit 17 was liquefied and injected upward along faults F4 and F5 throughout the sand layers of units 16 and 13, and terminated inside the sediment layer of Unit 12, indicating a faulting-liquefying event occurred during the deposition of Units 11 and 12. Such upward injection veins caused by paleoliquefactions have also reported in the seismogenic fault that triggered the 2010–2011 Canterbury earthquake sequence in Southwest Christchurch, New Zealand^18^.

At Loc. 1, the archaeological evidence reveals that the area inhabited during the Yayoi period was cut by normal faults with a vertical offset of 1.2 m^17^. The development of a graben structure buried numerous remains, including pottery fragments, firestones, and other artifacts^17^. The normal faults that cut the sedimentary layers formed at ~2760 yr B.P. and are covered by the younger layers that formed in the past 1600 years, indicating a graben-forming event that occurred during the Yayoi period, 1600–2600 yr B.P^17^. Therefore, we infer that this event resulted in the formation of the injection veins in units 12–17 at Trench A. Based on the timing constraint model, we infer that another faulting event (E3) occurred during the period between BC ~680 and AD ~200 (Event 3 in Fig. 6a).

In the lowest parts of the trench walls, the sand material of Unit 23 is disturbed by irregular veins and lenses showing brownish- to yellowish-color within a zone bounded by faults F4 and F5 in units of 19 and 20 and overlain by the sand layer of Unit 18 (Fig. 4; see Supplementary Fig. S4b–g). These observations indicate the liquefaction of a sand layer in Unit 23 and that the liquefaction event (E4) occurred in the period between Units 19–23 and Unit 18. Radiocarbon dating ages show that E4 occurred in the period between BC ~1200 and BC ~1000 (Event 4 in Fig. 6a).

## Discussion

Earthquakes with M ≥ 7 at shallow focal depths can generally produce distinctive coseismic surface ruptures and cause strong ground deformation that can be preserved in sedimentary horizons and therefore can be identified via trench surveys^19,20^. Two historical/paleoseismic events prior to the 2016 M_w_ 7.1 Kumamoto earthquake are identified on the HFFZ-Aso volcano system in this work; however, the magnitudes and epicenters of these earthquakes cannot be determined because no historically or instrumentally recorded earthquakes of M ≥ 6.5 have occurred within Aso caldera. Furthermore, there is no evidence of surface ruptures with significant offsets caused by the four largest foreshocks and aftershocks (M_w_ 6.0–6.5) that occurred in the two-week period surrounding the 2016 M_w_ 7.1 Kumamoto earthquake^4,21^.

Historical and paleoseismic studies have shown that the recurrence intervals of large earthquakes can be relatively well-constrained, thus providing the most direct measurements of recurrence intervals of moderate to large earthquakes along active faults^16,19,20^. Historical and instrumental records show that eight large earthquakes (6.7 ≥ M ≥ 6.0) have occurred in central Kumamoto Prefecture around the HFFZ (i.e., the present study area) in the past 400 years, and M ≤ 6.3 earthquakes occurred within Aso caldera in 1894 and 1895^15^. Historical earthquakes in the Japanese islands with magnitudes of <6.5 have not produced coseismic surface ruptures with distinct displacements and generally do not cause liquefaction^22^. Field investigations also confirm that the 2016 M_w_ 6.1 foreshock occurred on 14^th^ April 2016 produced neither distinct surface displacements nor earthquake-induced liquefaction within Aso caldera^3,4^. Therefore, we infer that the two M ≤ 6.3 historical earthquakes recorded in the past 400 years did not produce surface ruptures that altered local landforms and did not produce the liquefaction features discovered by our field studies. It follows that the three events before the 2016 earthquake that originated in this fault system also have magnitudes of ≥7.0, as this is the minimum magnitude necessary to produce the observed offsets, sedimentary layer properties, and liquefaction (Fig. 4). Therefore, we estimate an average recurrence interval of ~1000 years for large HFFZ earthquakes (Fig. 6b), consistent with previous estimates for the HFFZ^16^.

Two questions arise concerning the three large paleoearthquakes identified in this study. First, were the faulting events that offset the near-surface sediment layers triggered by faults within the caldera or in the HFFZ? Second, if the latter, did the seismic ruptures propagate northeastward across the caldera and terminate within it, as occurred during the 2016 M_w_ 7.1 Kumamoto earthquake? Geophysical and seismic studies have revealed that the crustal structures beneath Aso caldera are characterized by a low-velocity material (magma chamber) at depths of ~6 to 9 km^14,23–27^. Seismic inversion results suggest that up to 1−2 m of fault slip occurred at shallow depths (<6 km) along the seismogenic fault within Aso caldera, but no distinct slip occurred at depths of >6 km under the caldera^28–30^. Field observations also reveal that the coseismic slip distribution along the fault shows an asymmetry pattern and that the coseismic surface ruptures occurred mostly along the Kurokawa Fault and are dominated by normal faulting with a maximum vertical offset of 1.75 m^3,4^ (Fig. 2a and Supplementary Fig. S1). In the trench walls and outcrops, the vertical offsets of sedimentary units are observed, which are considered to be the component of net slips, because it is difficult to estimate the strike-slip component of motions within the caldera. Both ends of asymmetric fault slip distribution profile are considered to be initiation points of rupture and barriers where fault propagation is arrested^31^. These seismic inversion results and field observations demonstrate that the coseismic fault rupture propagated to the northeast near the surface, where coseismic surface ruptures were observed in the field, but stopped at the magma chamber under the caldera, at a depth of >6 km. It follows from first principles that neither coseismic faults nor fractures can develop in a magma chamber if the magma is in a liquid state^3^. Previous studies show that volcanic earthquakes can produce surface displacements accumulated on the faults that developed at shallow depths above the magma within the calderas^32,33^. In the study area, the Kurokawa Fault shows a straight linear trace, which also developed within Aso caldera. During the volcanic earthquake swarms occurred in November 1997 and March 1999 (Fig. 2b,c)^10^, there was no distinct surface displacement observed on this fault. Therefore, we conclude that the Kurokawa Fault developed at shallow depth of <6 km within Aso caldera did not have the potential to trigger large earthquakes.

Recent trench investigations of the Hinagu and Futagawa faults reveal that i) at least three morphogenic earthquakes prior to the 2016 Kumamoto earthquake occurring in the past 3000 years on the HFFZ (Fig. 6b), with the most recent event (E2) occurred in the period between AD ~850 and AD ~1150; ii) the penultimate morphogenic event (E3) took place in the period between BC ~80 and AD ~200; and iii) the third event (E4) occurred in the period between BC ~1600 and BC ~800^16^. The timings of the three paleoearthquakes identified in this study are highly consistent with previous trench-based study in the HFFZ, which assigned ages of AD ~1000, AD ~100, and BC ~1100 to events E2, E3, and E4, respectively (Fig. 6b). Accordingly, we suggest that the active faults of the HFFZ are the seismogenic faults that triggered the large paleoearthquakes; as in the case of the 2016 event. Coseismic rupture propagated northeastward to Aso caldera and displaced the near-surface sedimentary layers, finally terminating within the caldera (Fig. 2a). If the surface rupture lengths caused by these historical/paleoseismic earthquakes are the same as that of 2016 Kumamoto earthquake with a length of ~40 km^3^, the possible moment magnitude (M) is estimated to be ~7 by using the empirical relationship (M = 5.08 + 1.16 Log SRL) between the magnitude (M) and surface rupture length (SRL)^34^, comparable with the 2016 earthquake.

An earthquake cycle is likely to be stopped at the same boundary^35^. This boundary area in the HFFZ-Aso volcano system is characterized by high temperature and low shear wave velocity, density and resistivity, which may be related to the partially melted rheological condition at depths of 6 up to 15 km. This high-temperature material property near the volcano may act as a barrier to the dynamic rupture. Generally, the internal structure and local stresses within a fault zone can generate barriers to fracture propagation and contribute to fracture deflection and/or arrest^36^. In such cases, large faulting events in the earthquake cycle are stopped by the same barrier and produce identical magnitude as shown in this study. This study shows that large recurring earthquakes occur in an active fault-volcano system and that the magma chamber at Aso volcano played an important role in healing the propagation of seismogenic rupturing across the caldera.

## Conclusions

The following conclusions can be drawn on the basis of field investigations, trench excavations, and radiocarbon dating results:

1. Prior to the 2016 earthquake, at least three large normal faulting paleoearthquakes have occurred in the past ~3000 years, suggesting an average recurrence interval of ~1000 years for earthquakes large enough to produce surface ruptures along pre-existing normal faults within Aso caldera.
2. The most recent two large paleoseismic faulting events occurred in AD ~1000 and AD ~100, respectively.
3. Normal faulting paleoearthquakes within the caldera were the products of the combined HFFZ–Aso fault zone–volcano system, as was the 2016 M_w_ 7.1 Kumamoto earthquake.

Our results confirm that the volcano probably hinders the propagation of coseismic rupture during large earthquakes due to the presence of magma^3^.

## Electronic supplementary material

Supplementary figures
