# Supplementary material for: Recurrent large earthquakes related with an active fault-volcano system, southwest Japan
Source: Sci Rep. 2018 Sep 20;8:14081. doi: 10.1038/s41598-018-32140-8 (PMC6147789; doi:10.1038/s41598-018-32140-8)
Supplement: Supplementary file 1 — Supplementary figures [file 41598_2018_32140_MOESM1_ESM.docx]

***Supplementary information***

**Recurrent large earthquakes related with an active fault-volcano system, southwest Japan**

**Aiming Lin**^1^***, Peng Chen^1^ and Koichiro Sado^2^,**

^1^Department of Geophysics, Graduate School of Science

Kyoto University, Kyoto 606-8502, Japan

^2^Chiken Sogo Consultants Co. Ltd., Tokyo 116-0013, Japan

*******************************

***Corresponding author**

Dr. Aiming Lin

Department of Geophysics

Graduate School of Science

Kyoto University

Kyoto 606-8502, Japan

Email: [slin@kugi.kyoto-u.ac.jp](mailto:slin@ipc.shizuoka.ac.jp)

**Supplementary figures**


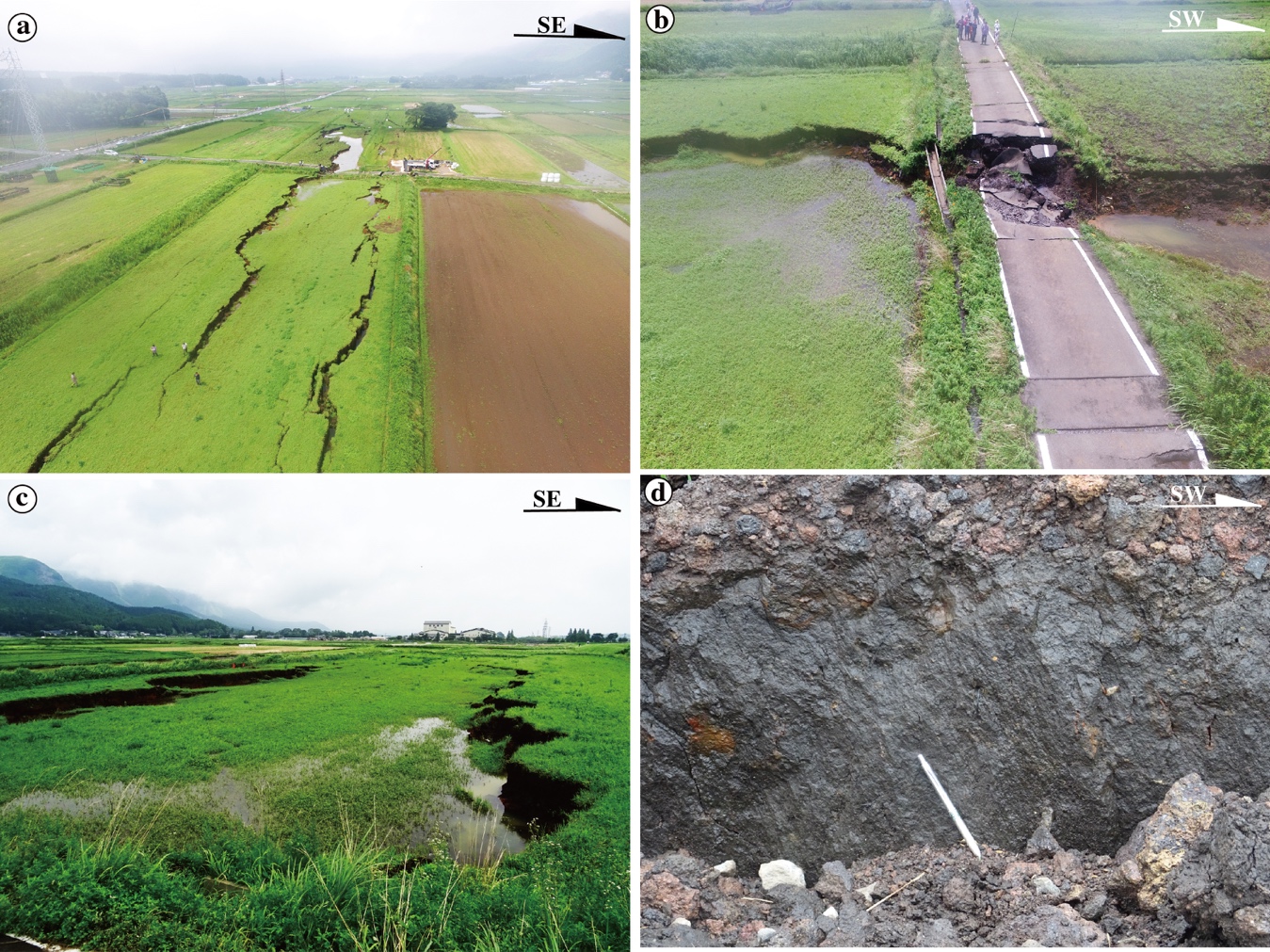


Figure S1. Representative photographs showing the coseismic graben structures produced by the 2016 Kumamoto earthquake. (**a**) and (**c**): Drone images showing the coseismic graben structures in a rice field^4^; (**b**) close-up view of (**a**), the road is vertically offset by 1.75 m^4^; (**d**) striations developed on the fault plane shown in (**b**) indicate a normal slipping sense.


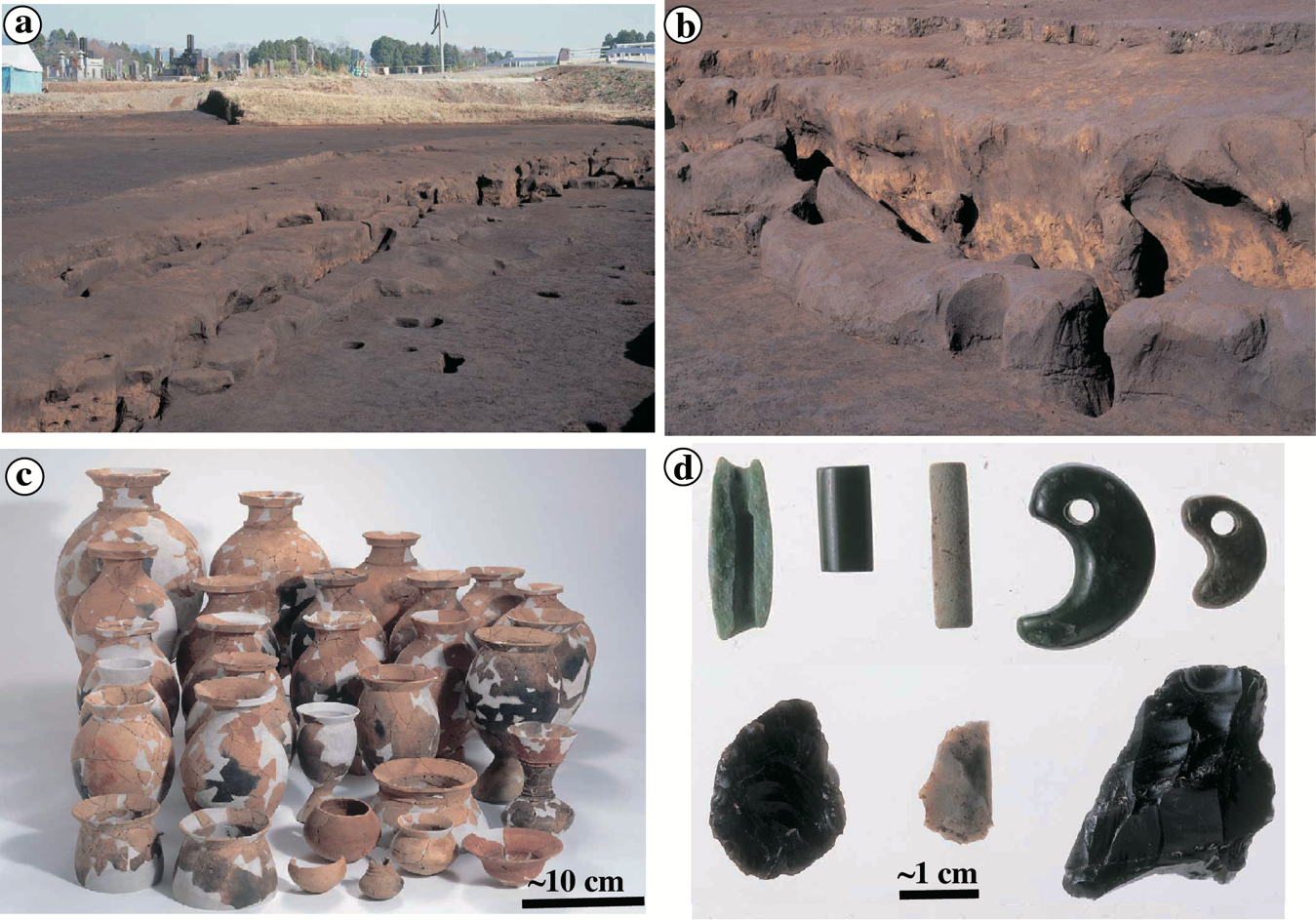


Figure S2. Graben structures exposed at an archaeological dig site (**a, b**) where pottery (**c**), jade, and stone implements (**d**) have been unearthed [cited from Education Committee of Kumamoto Prefecture (EDKP)]^22^.


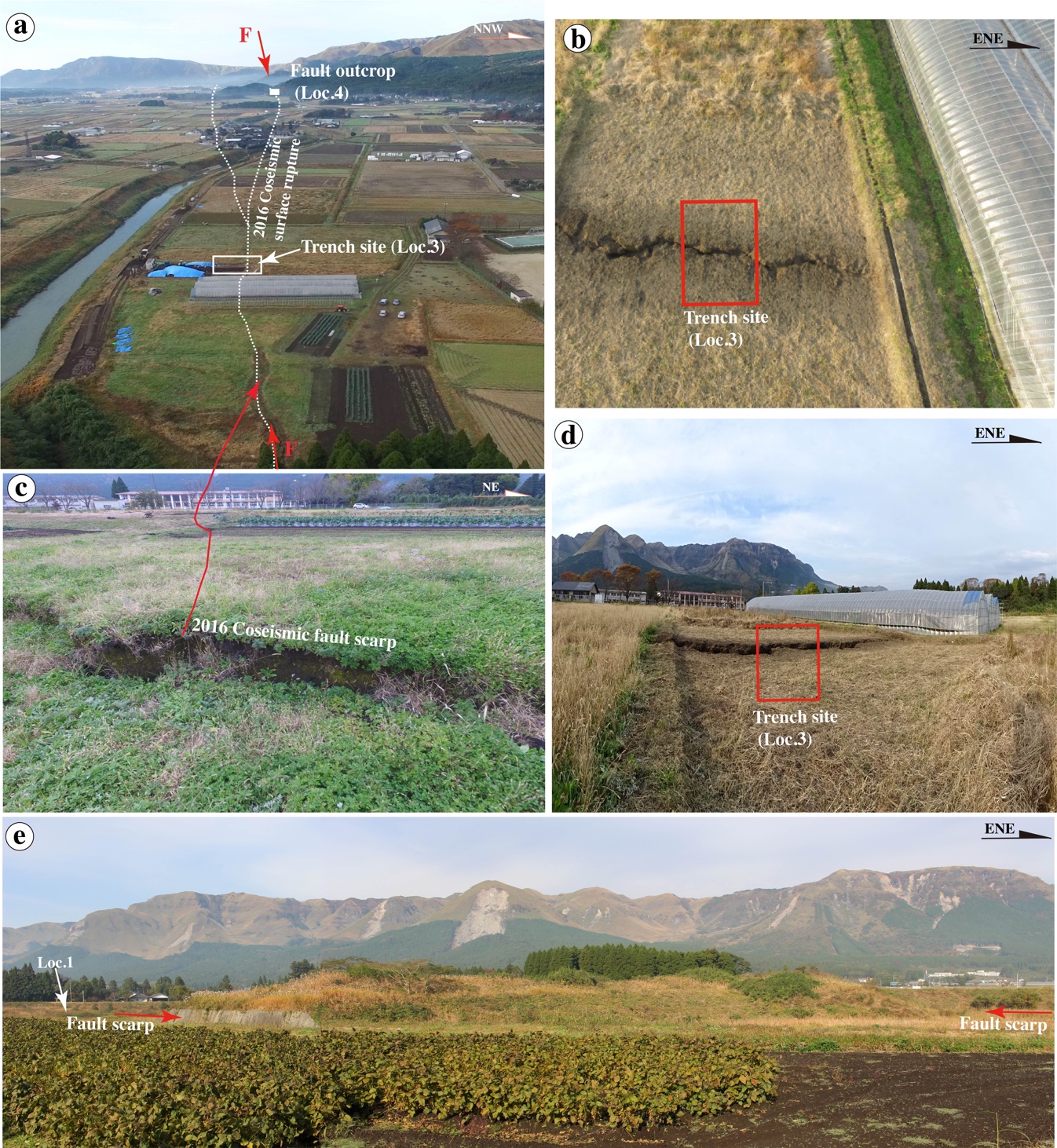


Figure S3. Representative photographs showing the coseismic surface ruptures produced by the 2016 Kumamoto earthquake in the study area where Trench A was excavated at Loc.3 across the rupture zone (a)-(d) and the fault scarp observed in Loc. 1 (e). (**a**)-(**d**): Drone images.


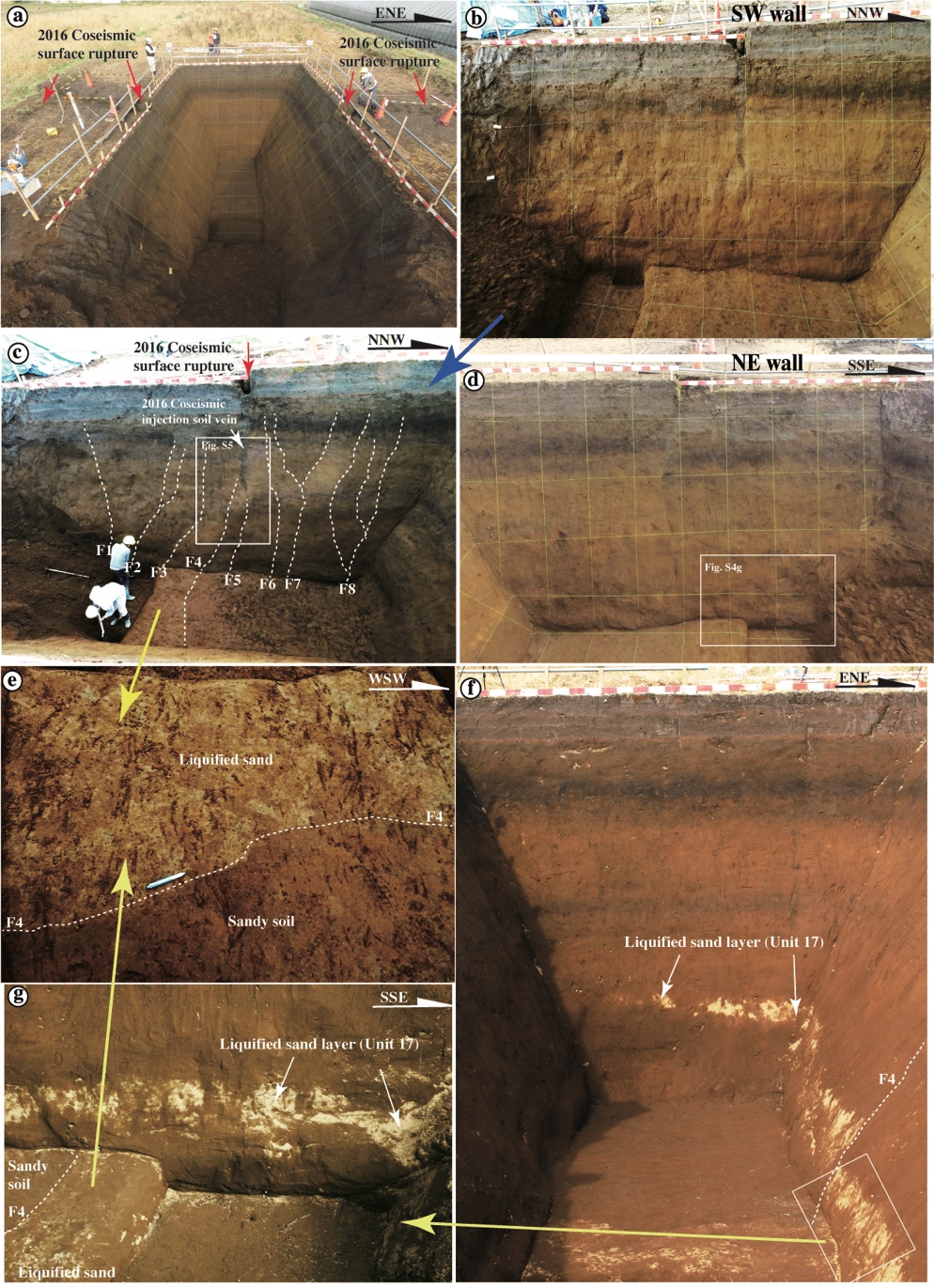


Figure S4. Photographs of trench exposure walls. (**a**) View of the entire trench (drone image); (**b**) southwest wall; (**c**) northeast wall; (**d**) faults in the southwest wall; (**e**) close-up view of the floor of the trench, where liquefied sand deposits occur as irregular veins and lenses (Unit 23); (**f**) liquefied sand layer of Unit 17 was offset by ~0.3 m along F4 fault; (**g**) close-up view of (f).

　　　　　
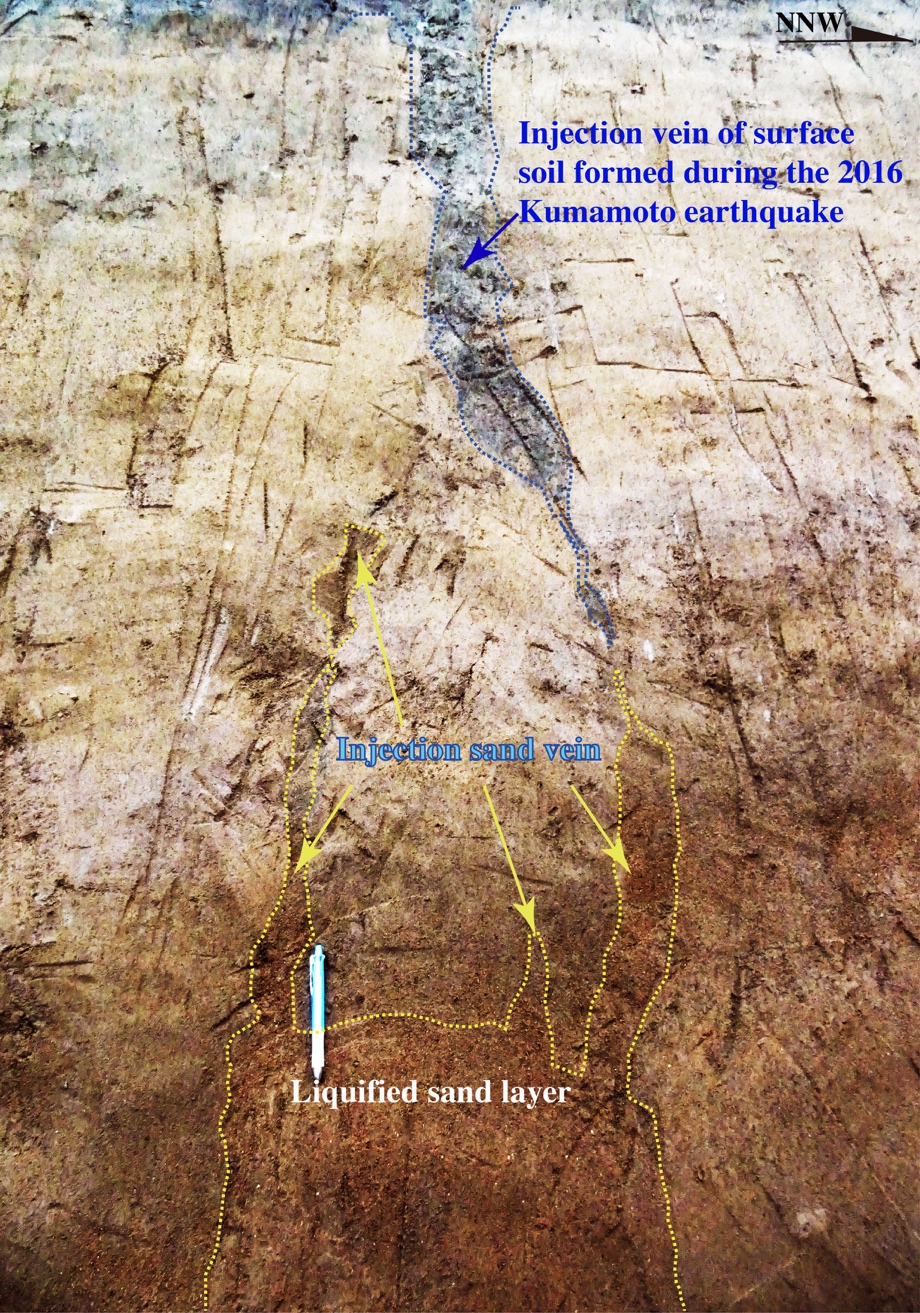


Figure S5. Close-up view of Figure S4b. The dark-gray surface soil material has been injected downward along the co-seismic fracture, whereas the brownish gray sand deposits have been injected upward and terminated at the end of the soil injection vein.
